# Preparation and Characterization of Novel Polyelectrolyte Liposomes Using Chitosan Succinate Layered over Chitosomes: A Potential Strategy for Colon Cancer Treatment

**DOI:** 10.3390/biomedicines12010126

**Published:** 2024-01-08

**Authors:** Asmaa Mokhtar Yosef, Raghad Saleh Alqarni, Fai Yahya Sayd, Manar Saleem Alhawiti, Raghad M. Almahlawi, Kousalya Prabahar, Ubaidulla Uthumansha, Mansuor A. Alanazi, Mohamed El-Sherbiny, Nehal Elsherbiny, Mona Qushawy

**Affiliations:** 1 Pharm. D Program, Faculty of Pharmacy, University of Tabuk, Tabuk 71491, Saudi Arabia; asmayousef135@gmail.com (A.M.Y.); raghadalqarni08@gmail.com (R.S.A.); faiy_2015@hotmail.com (F.Y.S.); manarselim.199@gmail.com (M.S.A.); ra.almahallawi@gmail.com (R.M.A.); 2 Department of Pharmacy Practice, Faculty of Pharmacy, University of Tabuk, Tabuk 71491, Saudi Arabia; 3 Department of Pharmaceutics, Crescent School of Pharmacy, B.S. Abdur Rahman Crescent Institute of Science and Technology, Chennai 600048, India; ubaidulla@crescent.education; 4 Department of Family and Community Medicine, Faculty of Medicine, University of Tabuk, Tabuk 71491, Saudi Arabia; menazi@ut.edu.sa; 5 Department of Basic Medical Sciences, College of Medicine, AlMaarefa University, Riyadh 13713, Saudi Arabia; msharbini@um.edu.sa; 6 Department of Anatomy and Embryology, Faculty of Medicine, Mansoura University, Mansoura 35516, Egypt; 7 Department of Pharmaceutical Chemistry, Faculty of Pharmacy, University of Tabuk, Tabuk 71491, Saudi Arabia; nelsherbiny@ut.edu.sa; 8 Department of Biochemistry, Faculty of Pharmacy, Mansoura University, Mansoura 35516, Egypt; 9 Department of Pharmaceutics, Faculty of Pharmacy, University of Tabuk, Tabuk 71491, Saudi Arabia; mqushawy@ut.edu.sa; 10 Department of Pharmaceutics, Faculty of Pharmacy, Sinai University, Alarish 45511, North Sinai, Egypt

**Keywords:** chitosomes, colon drug delivery, HT-29 colon cancer cells, liposomal assisted drug delivery, polyelectrolyte liposomes

## Abstract

Chitosan succinate is distinguished by its ability to shield the loaded drug from the acidic environment, localize and keep the drug at the colon site, and release the drug over an extended time at basic pH. The current study attempts to develop polyelectrolyte liposomes (PEL), using chitosan and chitosan succinate (CSSC), as a carrier for liposomal-assisted colon target delivery of 5 fluorouracil (5FU). The central composite design was used to obtain an optimized formulation of 5FU-chitosomes. The chitosan-coated liposomes (chitosomes) were prepared by thin lipid film hydration technique. After that, the optimized formulation was coated with CSSC, which has several carboxylic (COOH) groups that produce an anionic charge that interacts with the cation NH2 in chitosan. The prepared 5FU-chitosomes formulations were evaluated for entrapment efficiency % (EE%), particle size, and in vitro drug release. The optimized 5FU-chitosomes formulation was examined for particle size, zeta potential, in vitro release, and mucoadhesive properties in comparison with the equivalent 5FU-liposomes and 5FU-PEL. The prepared 5FU-chitosomes exhibited high EE%, small particle size, low polydispersity index, and prolonged drug release. PEL significantly limited the drug release at acidic pH due to the deprotonation of carboxylate ions in CSSC, which resulted in strong repulsive forces, significant swelling, and prolonged drug release. According to a 3-(4,5-dimethylthiazol-2-yl)-2,5-diphenyl-2H-tetrazolium bromide (MTT) assay, PEL treatment significantly decreased the viability of HT-29 cells. When compared to 5FU-liposome and 5FU-chitosome, the in vivo pharmacokinetics characteristics of 5FU-PEL significantly (*p* < 0.05) improved. The findings show that PEL enhances 5FU permeability, which permits high drug concentrations to enter cells and inhibits the growth of colon cancer cells. Based on the current research, PEL may be used as a liposomal-assisted colon-specific delivery.

## 1. Introduction

In recent years, liposome-assisted drug delivery has made significant progress in biomedical applications due to its benefits in improving cellular and tissue uptake, the biodistribution of drugs, facilitating site-specific delivery, and diminishing systemic toxic effects [1,2,3]. However, liposome has many issues such as inadequate physical and chemical stability, poor loading capability, prone to being broken down by gastric acid, pancreatic lipase, and intestinal bile salt at different parts of the gastrointestinal tract (GIT), and rapid elimination from blood circulation, leading to no guarantee that the drug will be absorbed into the cells and tissue at the colonic site [3,4]. To overcome the challenges, various strategies have been utilized for drug-encapsulated liposomes to effectively target the colon [5,6,7].

Surface modification of liposomes using natural and synthetic polymers has emerged as an attractive strategy for colon-target drug delivery [8,9]. Predominantly chitosan, pectin, and methacrylic acid polymers have been used to coat liposomes for enhanced colon target drug delivery through the oral route, via pH-dependent release in the GIT tract and strong mucoadhesive characteristics in colonic mucosa [10,11,12,13]. Jubeh et al. reported that charged liposomes have more adhesive power in colonic mucosa than neutral ones [14].

The natural cationic polysaccharide chitosan is widely utilized as an external coating on the liposomes. It is referred to as chitosomes, and its main mechanism of fabrication is the electrostatic interaction between the anionic charge of lipids and the positive charge of chitosan at acidic pH due to the presence of amino groups in the chitosan backbone chain. When compared to regular liposomes, chitosomes have enhanced drug uptake in colon tissue and higher stability in the stomach environment and intestinal contents [15,16]. However, due to its fast disintegration in the stomach and accumulation in the small intestine, its capacity to control the drug release in the gastrointestinal tract is constrained, and this leads to poor absorption in the colon [17,18]. To address this issue, colon-specific polymeric coating over chitosomes could be employed.

Chemically modified chitosan can overcome the constraints mentioned above in using plain chitosan, particularly in the context of controlled drug delivery, and expand the potential applications that can be achieved with chitosan [19]. Researchers are currently focusing on chitosan modification and its possible usage in drug delivery systems. Our ability to synthesize a variety of derivatives with various physicochemical properties is made possible by the reactivity of the primary amine groups in chitosan [20]. We selected the chitosan succinate (CSSC) as an outer coating polymer over chitosomes due to its earlier successful usage in designing colonic drug delivery systems [21]. The mechanism is that under acidic conditions, COOH groups in CSSC are nonionized and thus poorly hydrophilic, whereas, in basic conditions, COOH groups are ionized and hence become very hydrophilic [22]. This gives a basic idea to develop fluorouracil (5FU) loaded CSSC-coated chitosomes as a polyelectrolyte colon delivery system (PCDS) and to evaluate its anticancer efficiency.

The drug of choice for oral cancer, oropharyngeal cancer, colon rectal cancer, stomach cancer, and cervical cancer is 5 fluorouracil (5FU). The 5FU is classified as BCS class III, which is characterized by high solubility, low permeability, and strongly polar, exhibiting a pKa value of 8.0 [23]. It is poorly absorbed due to limited permeability, resulting in a low bioavailability (28%); additionally, it is rapidly eliminated after intravenous injection, with an apparent elimination half-life of 8–20 min. It has serious toxic side effects, including gastrointestinal, hematological, neurological, cardiac, and dermatological problems [24]. To improve 5FU oral absorption and therapeutic index, drug delivery systems such as liposomes have been intensively investigated [25,26].

In the current study, chitosomes were coated with CSSC to create a hybrid system designed to maximize the bioavailability of integrated 5FU and the rate at which it is released into the colon. To accomplish these objectives, it was essential to characterize the system thoroughly using various analytical instruments, including Fourier Transform Infrared Spectroscopy (FTIR), Light Scattering, zeta potential, and scanning Electron Microscopy (SEM). The prepared formulations were evaluated for the entrapment efficiency % (EE%), particle size, and in vitro drug release to obtain an optimized formulation of 5FU-chitosomes.

## 2. Materials and Methods

### 2.1. Materials

Chitosan and 5FU were obtained from Sigma–Aldrich (St. Louis, MO, USA). Cholesterol was received as a gift from Masterowin Pharmaceuticals (Chennai, India). Tween 80 and phosphatidylcholine were purchased from Spectrum Chemical (New Brunswick, NJ, USA). Succinic anhydride was given from Merck (Mumbai, India). The distilled water came from a source within the organization. All the remaining reagents and chemical substances used in the investigation were of analytical grade.

### 2.2. Design of the Experiment (DOE)

The response surface methodology has been applied to construct and design optimal formulation of 5FU-chitosomes as a potential colon delivery system. The Central Composite Design method was utilized to optimize the parameters that comprise the 5FU-chitosomes formulation using Design Expert Software version 11 (Stat-Ease, Minneapolis, MN, USA) (https://www.statease.com/docs/v11/ accessed on 28 December 2023). Three formulation factors (independent variables), cholesterol (X1), chitosan (X2), and surfactant (X3) were utilized. The effect of the independent variables was studied in three responses (dependent variables), entrapment efficiency (Y1), particle size (Y2), and drug release % (Y3) to determine the best formulation. The experimental design contained a randomized sequence, six instances of replication with central points, six axial points, and eight designated factorial points. Five iterations of the central point were performed to assess the repeatability of the method. To assess the data, a technique known as response surface regression was applied. When choosing a polynomial model, the significant terms (*p* < 0.05), least significant lack of fit, coefficient of variance, and multiple correlation coefficients provided through the Design Expert program were considered. The highest level and lowest levels associated with the independent variables are shown in Table 1.

### 2.3. Preparation of Chitosomes

Cholesterol and Tween 80 along with phosphatidylcholine were mixed in an organic solvent mixture of methanol and chloroform in a rounded bottom flask. The organic solvent was allowed to be evaporated to obtain a thin lipid film in the wall of the rounded bottom flask. The dried film was hydrated with phosphate buffer containing 5FU. The liposomal dispersion was sonicated to reduce the particle size. Chitosan powder dissolved into a 0.5% *v*/*v* acetic acid solution with continuous stirring to prepare the 1% *w*/*v* chitosan solution needed to provide the coating material. Chitosan solution was added dropwise to the liposomal dispersion while sonicated. The resulting dispersion was continuously stirred for two hours at room temperature. Negatively charged liposomes and positively charged chitosan interacted electrostatically to form the chitosan-coated liposomes (chitosomes).

### 2.4. Synthesis of Chitosan Succinate (CSSC)

CSSC was prepared based on the earlier report [22]. CSSC was prepared by dissolving 1 g of each chitosan and succinic anhydride in 20 mL of DMSO. The obtained mixture was stirred at 1000 rpm for 6 h and kept at 63 ± 5 °C. The pH of the solution was raised to five by adding NaOH solution (7% *w*/*v*). The precipitate was collected and dissolved in 100 mL of water, followed by adjusting the pH of the solution to 10 using NaOH solution, producing a yellow-colored solution. Following three acetone washes, the solution was recrystallized to yield N-succinyl-chitosan, which was then lyophilized to form a pale yellow powder. It was then stored in well closed container until needed to be used again.

### 2.5. Infrared (IR) Spectroscopy

The chitosan and CSSC polymers were investigated by employing infrared spectroscopy. Each sample was compressed into discs using potassium bromide (KBr). Each disc was scanned in the range of 4000 cm^−1^ to 400 cm^−1^ using a Thermo Scientific Nicolet IR 200 spectrometer (Thermo Scientific, Waltham, MA, USA) [27].

### 2.6. Preparation of Chitosan Succinate-Coated Chitosomes (PEL)

CSSC solution of 2.5% *w*/*v* was prepared by dissolving CSSC using distilled water and then added gradually to an equal volume of the previously prepared 5FU-chitosomes dispersion at room temperature, with stirring at 100 rpm for about 2 h using a magnetic stirrer. The pH of the prepared CSSC-coated chitosomes was adjusted to five before being stored overnight at 4 °C in the refrigerator. The final product was referred to as PEL.

### 2.7. Evaluation of Properties of 5FU-Chitosomes

#### 2.7.1. Entrapment Efficiency % (EE%) of 5FU-Chitosomes (Y1)

An aliquot (5 mL) of each formulation was centrifuged at 10,000 rpm for 15 min to determine the EE%, applying an indirect analysis method [28,29]. To quantify the free 5FU, the clear supernatant was estimated by using a UV spectrophotometer at 266 nm. The EE% of 5FU was calculated using the following equation.

$$EE\%=\frac{Total\;amount\;of\;5FU-Unentrapped\;5FU}{Total\;amount\;of\;5FU}\times 100$$

#### 2.7.2. Determination of Particle Size of 5FU-Chitosomes (Y2)

The particle size and polydispersity index of the prepared 5FU-PEL were determined by dynamic light scattering technique using Malvern^®^ Zetasizer Nano ZS90 (Malvern^®^ Instruments Limited, Worcestershire, UK). The prepared formulations were diluted with double distilled water and subjected to measurement at room temperature and an angle of 90° [30]. All samples were measured in triplicate and mean ± SD was determined.

#### 2.7.3. In-Vitro Release Study of 5FU-Chitosomes (Y3)

The in vitro release study was conducted using the dialysis bag technique. Samples were added in dialysis bags with the ends cut off (12–14 kDa), and submerged into the dissolving media, which was heated to 37 ± 0.5 °C. For a total of 24 h, the release of 5FU was observed (initial 2 h with Simulated Gastric Fluid (SGF); pH 1.2 and rotation of basket set at 200 rpm, followed by Simulated intestinal fluid (SIF); and a pH 7.4 solution throughout rotation at 100 rpm). To ensure sink conditions, 1 mL buffer solution was removed and replaced with a new buffer medium at predetermined intervals. The amount of 5FU released was determined by a spectrophotometer with a UV-visible wavelength of 266 nm. Different kinetic equations, such as the zero-order, first-order Higuchi, Hixson–Crowel, and Korsmeyer–Peppas equations, were used to analyze the in-vitro dissolution data. The linear curves derived by regression analysis of the plots were given coefficients of correlation (r^2^) and constant (k) values. The Ritger and Peppas model was used to create the release mechanism using the release data gained from the aforementioned approach. The diffusion exponent ‘n’ was estimated using the following equation utilizing the initial 60% cumulative release data.

MtM∞=Ktn

where, at time t, is the amount of medicine released M_t_, M_∞_ is the notional total amount released, K is the kinetic constant, and n is the diffusion exponent used to describe the release mechanism. A value of n 0.43 for spheres suggests Fickian release, while a value of n between 0.43 and 0.85 shows non-Fickian release (both diffusion-controlled and swelling-controlled drug release). A case-II transport with a ‘n’ value of less than 0.85 involves polymer breakdown and polymeric chain elongation or relaxation.

#### 2.7.4. The Optimization Process

The prepared 5FU-chitosomes formulations were subjected to numerical optimization using Design Expert Software version 11. The selection of optimized formulation is based on maximizing the EE%, minimizing the particle size, and prolonging the drug release. The optimized formulation of 5FU-chitosomes was prepared and evaluated for EE%, particle size, zeta potential, in-vitro release, and mucoadhesive properties in comparison with equivalent 5FU-liposomes and 5FU-PEL. The EE%, particle size analysis, and invitro release were determined as mentioned before. To investigate the physical stability of the developed 5FU-liposomes, 5FU-chitosomes, and 5FU-PEL formulations, the Zeta Potential was evaluated. Samples of each formulation were diluted and analyzed by keeping them in an electrode cell container. A zeta analyzer was used to measure the zeta potential of the formulations [31].

#### 2.7.5. Scanning Electron Microscopy (SEM) of 5FU-PEL

SEM was used to determine the shape and surface properties of the optimized 5FU-PEL by gold sputter technique using Tescan Vega 3 scanning electron microscope (Tescan Company, Brno, Czech Republic). The optimized 5FU-PEL was sprinkled onto an aluminum stub with double-sided tape. The sample was then coated with gold to a thickness of 400 A° using a chilled sputter coater. At an accelerated voltage of 20 kV and a chamber pressure of 0.6 mmHg, photomicrographs were taken.

#### 2.7.6. Swelling Index of 5FU-PEL at Different pH Conditions

The swelling property of the 5FU-PEL was investigated in acidic and basic pH conditions. PEL (1 g) was weighed and put into a 100 mL measuring container with 10 mL of each pH 1.2 and 7.4 buffers. The measurement of the initial weight (W_i_) of the dried sample was recorded, and a variation on the actual weight of the wet sample (W_t_) with a maximum duration of 6 h later was observed. The formula that follows was used to determine the swelling intensity.

$$SI\;(\%)=\frac{Wt-Wi}{Wi}\times 100$$

#### 2.7.7. Mucoadhesive Study of 5FU-PEL

The mucoadhesive properties were evaluated by an in-vitro wash-off test. The rat specimen was tied onto a glass slide using thread. Samples of 5FU-liposomes, 5FU-chitosomes, and 5FU-PEL were spread onto the wet, rinsed, specimen, and allowed to hydrate for 30 s. The prepared slide was hung onto one of the grooves of a USP 24-tablet disintegrating test apparatus. The disintegrating test apparatus was operated in such a way that the specimen was given regular up and down movements in the vessel containing one liter of pH 7.4 buffer at 37 °C. At the end of 8 h, the apparatus was stopped and the mucoadhesive strength was evaluated using the following equation:

$$MS\;(\%)=\frac{Total\;amount\;PEL\;adhered}{Total\;amount\;of\;PEL\;added}\times 100$$

#### 2.7.8. Stability Study

A sample of the 5FU-PEL formulation was stored in a glass bottle and kept inside the stability chamber through different temperatures, including room temperature (25 °C) with a relative humidity of 60% and refrigeration (4 °C). The sample was examined for the drug content and the particle size at zero, and 6-month time intervals.

#### 2.7.9. In Vitro Cytotoxicity Test

The cytotoxicity of 5FU-liposomes and 5FU-PEL were determined by 3-(4,5-dimethylthiazol-2-yl)-2,5-diphenyl-2H-tetrazolium bromide (MTT) assay against HT-29 colon cancer cell lines. The MTT assay is used to assess cell viability, proliferation, and cytotoxicity by measuring cellular metabolic activity. Surviving cell numbers were determined by MTT dye reduction, water soluble tetrazolim dye was reduced by live cells to a purple formazan. HT-29 cell lines were treated with samples and plates were gently shaken and incubated for 4 h at 37 °C in a 5% CO_2_ atmosphere. The supernatant was removed, 100 µL of propanol was added and plates were gently shaken to solubilize the formed formazan. The absorbance of the samples was measured at 540 nm using a microplate reader (Model No 680XR reader Bio-Rad Laboratories, Hercules, CA, USA). The following formula was used to calculate the cell viability.

$$Cell\;viability\;\left(\%\right)=\frac{\left(OD\;sample-OD\;blank\right)}{\left(OD\;control-OD\;blank\right)}\times 100$$

### 2.8. In Vivo Pharmacokinetics Study

Male Wistar rats (230–250 g) were obtained from the Central Animal House of C.L. Baid Metha College of Pharmacy, Chennai, India. The animals were kept under standard laboratory conditions, with the temperature at 25 ± 1 °C and relative humidity of 55% ± 5%. The animals were accommodated in polypropylene cages, four per cage, with free access to a standard laboratory diet (Lipton feed, Mumbai, India) and water ad libitum. The protocol of the study was approved by the Institutional Animal Ethics Committee (approval number 15/322/PO/Re/S/01/CPCSEA).

Groups of rats (each of 06) were injected intraperitoneally with a single dose of 5FU-liposomes, 5FU-chitosomes, and 5FU-PEL (at 5FU equivalent dose 20 mg/kg). Blood samples were collected from the rats at different time intervals 0, 1, 2, 4, 6, 8, 10, 12, 16, 20, and 24 h after dosing. The blood samples were collected in tubes containing ethylenediaminetetraacetic acid (EDTA) as an anticoagulant and immediately centrifuged at 3000 rpm for 15 min. The separated plasma samples were stored at −20 °C until analyzed. A High-performance liquid chromatography (HPLC) approach was used to quantify the amount of 5FU in the samples with slight modification of earlier methods [32]. The mobile phase consisted of 40 mM phosphate buffer, which was adjusted to pH 7.0 using 10% *w*/*v* potassium hydroxide. The sample was injected into the HPLC system (Waters^TM^, Milford, MA, USA) using a 15-μL volume that was pumped through a C_18_ column at a rate of 1 mL/min. For each sample, 5FU was detected using a UV/Vis detector set to 260 nm. Plasma concentration–time curves of 5FU were evaluated using non-compartmental analysis. The values of maximum plasma concentration (C_max_) and the time to C_max_ (T_max_) were directly obtained from experimental observations. The elimination rate constant (ke) was obtained from a log-linear regression analysis of the plasma concentration–time curves in the elimination phase. The elimination half-life (t_1/2_) was calculated from k_el_. The area under the concentration–time curve from 0 h to the last quantifiable concentration (AUC_0–t_) was calculated by the linear trapezoidal method. The area from the last measured concentration to infinity (AUC_t–∞_) was calculated by dividing the last measurable plasma concentration by the k_el._ The mean residence time (MRT) and relative bioavailability also were calculated [33,34].

### 2.9. Statistical Analysis

The results of the in vivo study were subjected to statistical analysis using the GraphPad Prism statistical package. Student’s *t*-test was used and the difference was considered significant at *p* values < 0.05.

## 3. Results and Discussion

### 3.1. FTIR Spectra of Chitosan and Chitosan Succinate Polymer

**Figure 1:**
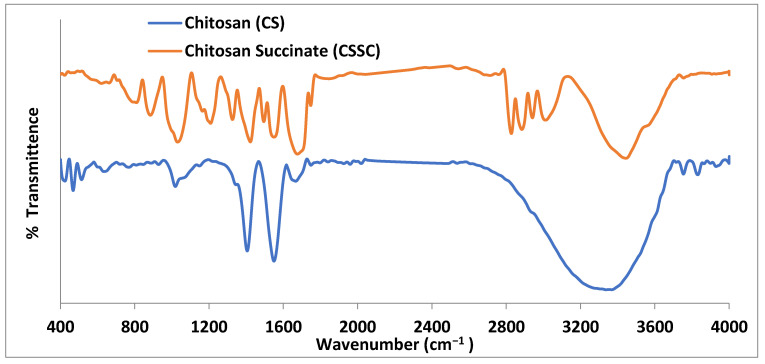
FTIR spectra of chitosan and chitosan succinate.

FTIR spectra belonging to chitosan and CSSC are shown in Figure 1. Chitosan absorption was distinguished by a band at 1570.13 cm^−1^, attributable to the stretching of the vibration of the amino group, and 1432.64 cm^−1^, related to the C-H vibration. The stretching vibration generated by the amine NH causes a second band at 3440.13 and the peak at 2925.6 cm^−1^ appears characteristic of C-H vibration. The saccharide structure of chitosan is represented by the peaks between 891.2 and 1158.1 cm^−1^. The broad peak at 1095.1 cm^−1^ indicates the stretching vibration belonging to C-O. In addition to the peaks mentioned above, The IR spectra of CSSC also revealed a particular peak starting at 1653.8 cm^−1^, which is attributed to carboxylic moieties’ C-O stretching vibrations. The peak of the signal around 1639.3 cm^−1^ suggested that C-O vibrations associated with the stretching of carboxylic moieties occurred [29]. The outcomes thus support the presence of carboxylic moieties connected with the chitosan backbone network that is present in CSSC polymer [35].

### 3.2. Optimization of 5FU-Chitosomes

In the present study, response surface methodology using Stat-Ease Design Expert software version 11 was used for the preparation of the 5FU-chitosomes. The central composite design was used to study the key formulation variables for the experimental design that influence the responses of entrapment efficiency (Y_1_), particle size (Y_2_), and drug release for 12 h (Y_3_) of the chitosomes. The experimental study was carried out to investigate the effect of the concentration of cholesterol, concentration of chitosan, and surfactant concentration on the above responses. According to the central composite design, a total of 20 formulations were prepared in a three-factor, three response set up with six center points. Further, the responses were evaluated using Analysis of variance (ANOVA) and their respective response surface methodology plots to find the influence of various factor combinations on the responses. The three-factor central composite design matrix produced by the software and the experimental data are summarized in Table 2.

### 3.3. Study of Effect of Formulation Factors (X_1_, X_2_, X_3_) on Responses (Y_1,_ Y_2_, Y_3_)

#### 3.3.1. Effect of the Independent Variable on Entrapment Efficiency (EE%) (Y1)

As shown in Table 2, the EE% of the prepared 5FU-chitosomes ranged from 20 ± 1.88% to 89 ± 2.02%. The EE% was highly affected by the formulation factors. The interactions between the independent factors were explored on the EE when negatively charged lipids and positively charged chitosan electrostatically interacted to generate chitosan-coated liposomes (chitosomes).

**Figure 2:**
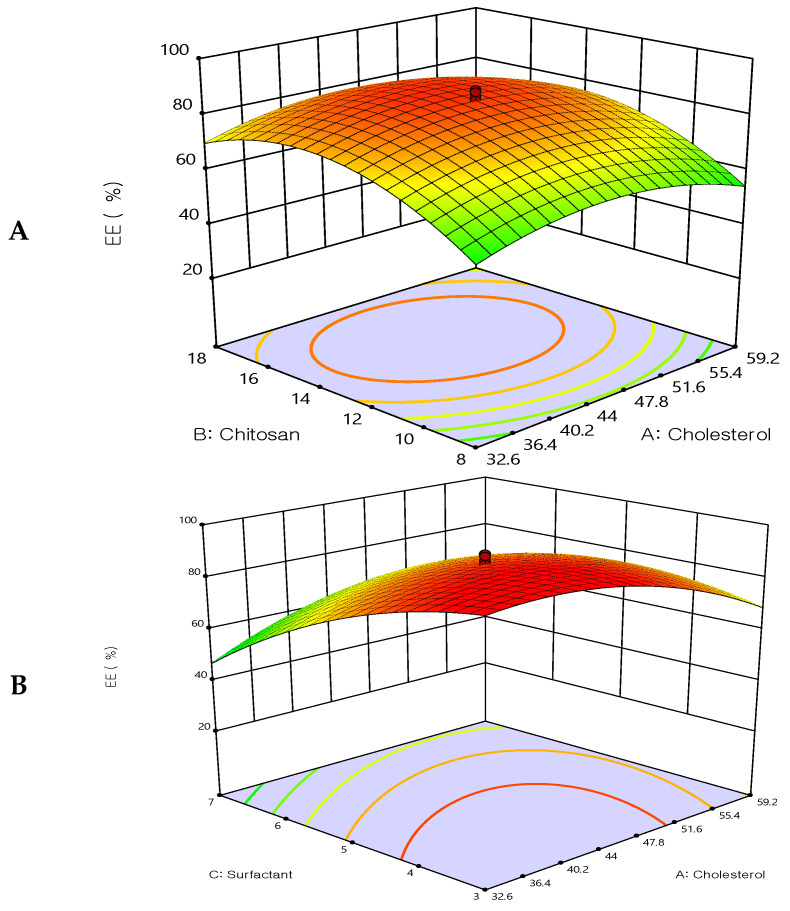

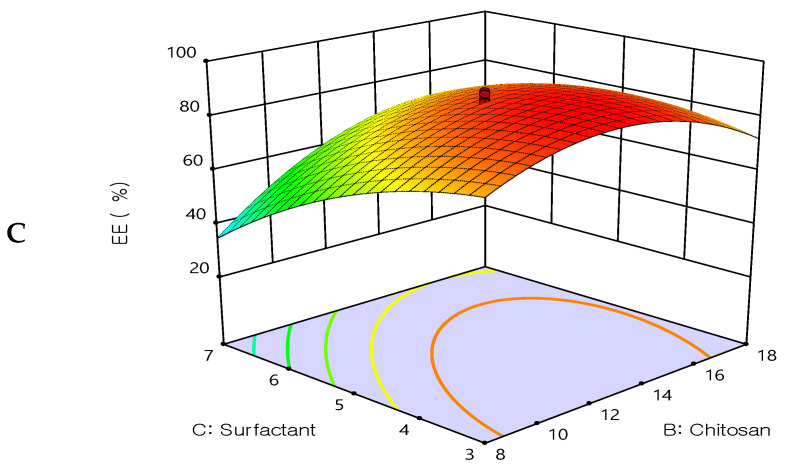
Three-dimensional surface plot for the influence of the independent variables on Entrapment Efficiency. (**A**) Effect of cholesterol and chitosan on EE%, (**B**) Effect of cholesterol and surfactant on EE%, (**C**) Effect of chitosan and surfactant on EE%.

The response surface methodology linked with the effectiveness of drug entrapment showed that increasing the concentrations of cholesterol and chitosan led to an improvement in the EE% (Figure 2). The concentration of cholesterol has a linear relationship with entrapment efficiency, cholesterol is found to improve membrane fluidity thereby enhancing the distribution of aqueous phase within the liposomal vesicles. Due to chitosan adhering to the outermost layer of the liposomes, which increased the drug’s holding power, the prepared formulations had a considerably improved EE%. These findings are in agreement with earlier studies [36,37], which revealed that when the chitosan concentration was increased, there was no longer any drug leakage since the equilibrium of chitosan adsorption to the liposome surface had been reached. The entrapment efficiency was decreased by increasing Tween 80. These results may be due to Tween 80 being composed of a chain of unsaturated alkyls. The presence of double bonds caused the chains to bend, resulting in the creation of an inadequately tight liposomal membrane. Therefore, the membrane of the liposomes was more permeable, which may account for the reduced entrapment efficiency of the Tween 80 formulations. Additionally, a report has indicated that the entrapment efficiency was increased by increasing the cholesterol content.

The quadratic polynomial equations expressed the effect of formulation factors in EE%:

EE% = 85.16 − 0.495908X_1_ + 7.30165X_2_ − 11.3662X_3_ + 0.125X_1_X_2_ + 10.375 X_1_X_3_ + 9.875X_2_X_3_ − 10.2423X_1_^2^ − 15.1213X_2_^2^ − 9.18165X_3_^2^

#### 3.3.2. Effect of the Independent Variable on Particle Size (Y2)

All prepared formulations were evaluated for the particle size analysis by dynamic light scattering technique. The particle size results were found in the range from 3.4 ± 0.2 μm to 32.4 ± 0.5 μm, see Table 2.

**Figure 3:**
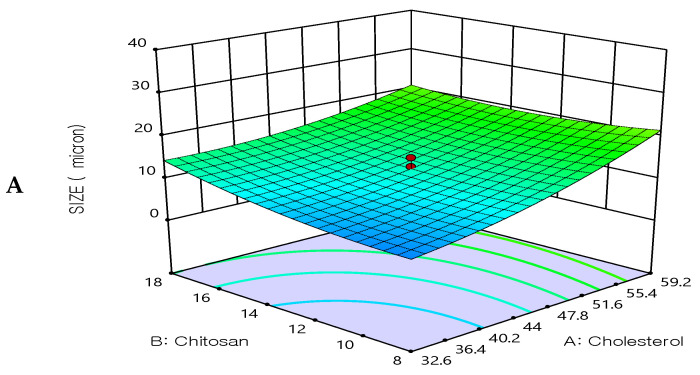

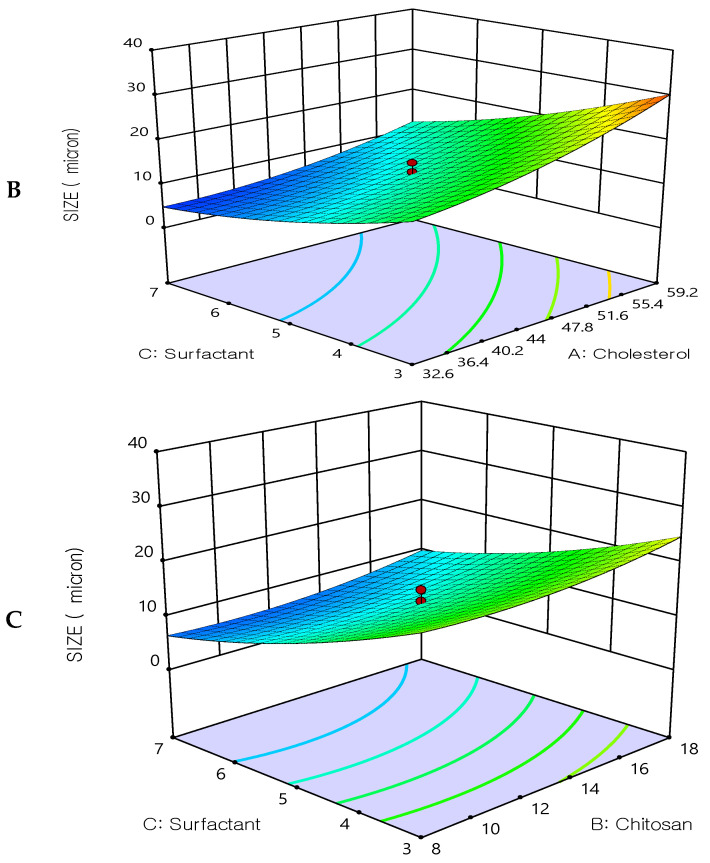
Three-dimensional surface plot for the influence of the independent variables on Particle size. (**A**) Effect of cholesterol and chitosan on particle size, (**B**) Effect of cholesterol and surfactant on particle size, (**C**) Effect of chitosan and surfactant on particle size.

The optimization study’s objective aimed to minimize the size of the prepared 5FU-chitosomes. Three-dimensional response surface plots serve as evidence that the impacts caused by the independent variables with the size were investigated (Figure 3). The 5FU-chitosomes containing Tween 80 were found to be in smaller particle size which may be due to the reduction in surface tension, resulting in phospholipid uniform arrangement in small vesicles [32]. Increasing the concentration of cholesterol resulted in increases in the particle size. An increase in chitosan content resulted in increased particle size whereas, above a certain concentration, rearrangement of the lipid bilayer led to the formation of small particle-sized vesicles. A smaller particle size was found because complete liposome coating/restabilization is guaranteed by a great chitosan polymer content in conjunction with ideal coating process conditions. However, there are situations when a discernible rise in size is seen after coating with a relatively small amount of chitosan solution, which can be explained by the aggregation of two or more vesicles that are tightly packed together [38,39]. The polydispersity index for all prepared 5FU-chotosomes was less than 0.5 which indicates the homogeneity of size distribution. The quadratic polynomial equations expressed the effect of formulation factors in particle size:

Particle size = 12.47 + 5.20435X_1_ + 1.64638X_2_ − 7.53829X_3_ − 1.9525X_1_X_2_ − 1.5525 X_1_X_3_ + 0.0475X_2_ X_3_ + 2.23154X_1_^2^ + 1.87798X_2_^2^ + 1.8992 X_3_^2^

#### 3.3.3. Effect of the Independent Variable on Drug Release (Y3)

**Figure 4:**
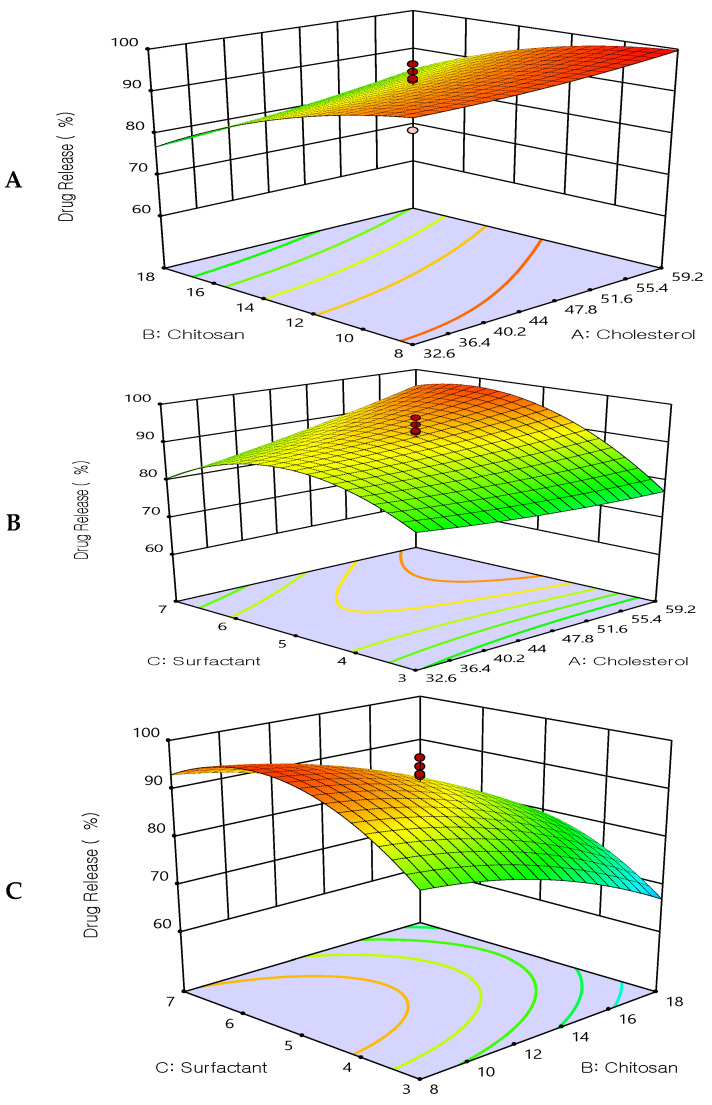
Three-dimensional surface plot for the influence of the independent variables on Drug Release. (**A**) Effect of cholesterol and chitosan on drug release %, (**B**) Effect of cholesterol and surfactant on drug release %, (**C**) Effect of chitosan and surfactant on drug release %.

All prepared formulations were studied for the in vitro drug release using a dialysis bag. It was found that all formulations exhibited slow, extended drug release. The drug release after 12 h ranged from 60.25 ± 2.6% to 99.89 ± 2.4%, see Table 2. The extended drug release may be due to the enclosed lipid shell of liposomes, which permits a slower release of 5FU from the lipid matrix. Figure 4, shows 3D response-surface plots showing the influence of the formulation factors on the drug release Q_12 h_ (Y_3_). The release of 5FU extended up to 12 h when liposomes were coated with chitosan. At pH 7.4, the amount of 5FU released tended to decrease as the number of liposomal layers increased, indicating that the polymer layers regulate and delay the drug’s release. However, the drug release was increased as the surfactant increased (Tween 80). These results may be due to the hydrophilicity of the used surfactant and the smaller size of the prepared vesicles, which resulted in higher surface area and hence improved the drug release.

The quadratic polynomial equations expressed the effect of formulation factors in the drug release:

DR_12 h_ = 91.62 + 2.79135X_1_ − 8.54936X_2_ + 4.47318X_3_ + 1.06875X_1_X_2_ + 4.58375X_1_X_3_ − 0.41625X_2_X_3_ − 0.255803X_1_^2^ − 4.13428X_2_^2^ − 10.0033 X_3_^2^

### 3.4. Optimization of Formulation Factors

A quadratic model that relates the responses and the independent variables was chosen by the central composite design and described in terms of the coded parameters by the second-order polynomial equations. The updated model’s multiple correlation coefficient (R^2^) and confidence interval (P) were used to evaluate it. As represented in Table 3, the equations of (EE), (size), and DR all had R^2^ values of 0.9707, 0.9690, and 0.9718, respectively. A good fit model is defined as an R^2^ value of more than 0.80, implying that the applied regression model is adequate [40]. This implies that the process parameters investigated explain more than 98% of the variability in chitosome characteristics and that the model could not explain only 2% of the variation in response [41].

The lack of fit (*p* > 0.05) was not significant for any of the fitted models (Table 4, Table 5 and Table 6). Non-significant lack of fit is beneficial for the model to fit in response surface methodology [42]. As a result, the chosen model may be applied to the simulation and optimization of variables for chitosome preparation.

**Figure 5:**
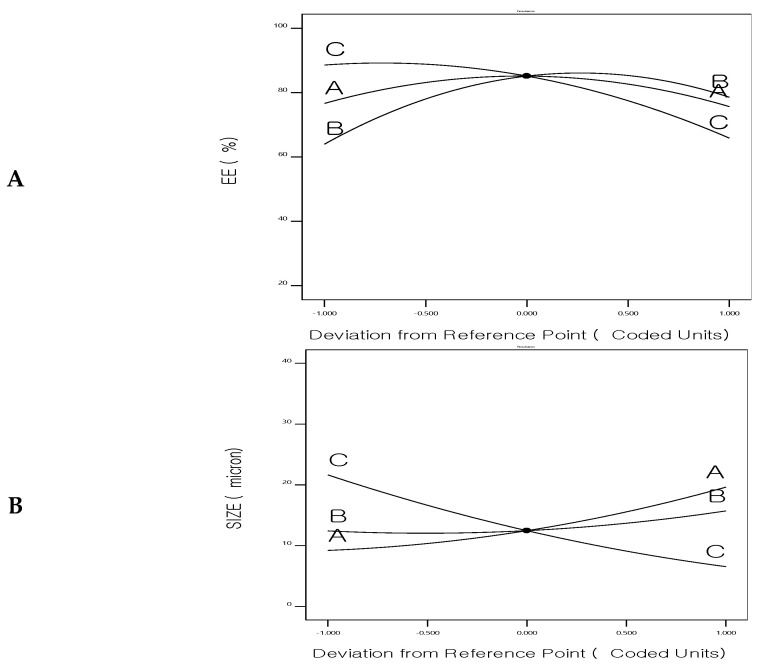

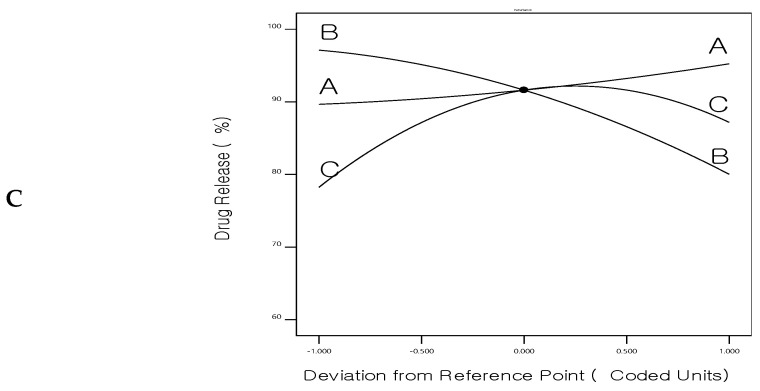
Perturbation chart for (**A**) Entrapment efficiency%, (**B**) Size, and (**C**) Drug release of 5FU-chitosomes.

Figure 5 shows the main-effect graphs for the influence of the formulation factors on the EE%, particle size, and drug release of 5FU-chitosomes. Numerical optimization, utilizing a central composite design, was applied to obtain optimized 5FU-chitosomes with high EE%, small particle size, and prolonged drug release. So, using Design Expert 11 software, a constraint was chosen for each answer to obtain the best level associated with each formulation factor, resulting in an optimally designed formulation.

**Figure 6:**
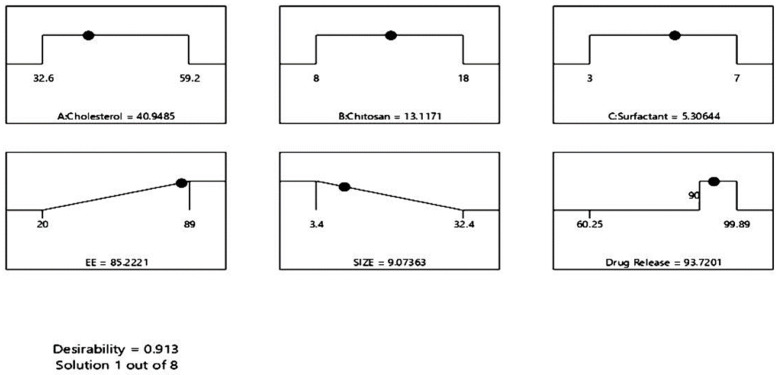
Ramp plot of optimal formulation factors and responses of 5FU-chitosomes.

The ideal formulation factors for the optimized formulation of 5FU-chitosomes according to the central composite design are shown in Figure 6. The composition of the optimized formulation was found that 40.9485 mg of cholesterol, 13.1171 mg of chitosan, and 5.30644 mg of surfactant with predicted values that are 85.221% for EE%, 9.07 μm for particle size and 93.7201% for drug release (Q_12 h_)_._

**Figure 7:**
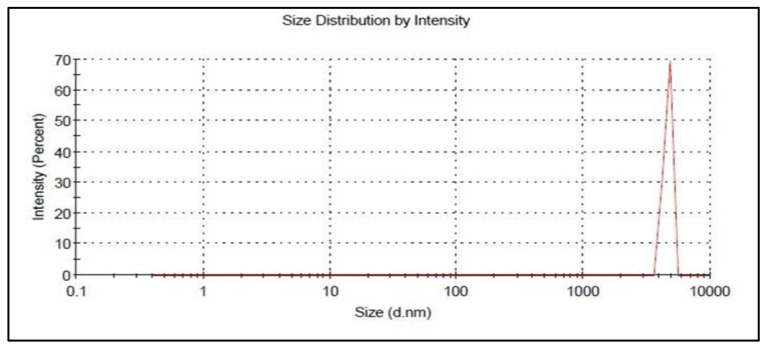
Particle size of the optimized 5FU-chitosomes.

The optimized 5FU-chitosomes were prepared based on the values obtained by numerical optimization using Design Expert Software. The prepared formulation was evaluated for EE%, particle size, and the in vitro drug release. The optimized 5FU-chitosomes showed 83.56 ± 2.37% for EE%, 9.3 ± 0.65 μm for particle size, and 95.16 ± 3.74% for the drug release, See Table 7 and Figure 7. The predicted and observed values of the dependent variables for the optimized formulations, as well as the percentage error resulting from the responses, were presented in Table 7. The percentage error is quite low because the values that were observed were remarkably comparable when compared with the predicted values, indicating the validity and reliability of the central composite design employed for chitosome optimization (desirability index = 0.913).

### 3.5. Evaluation of 5FU-Polyelectrolyte Liposomes (5FU-PEL)

#### 3.5.1. Entrapment Efficiency (EE%)

The EE% values of 5FU-liposomes and 5FU-chitosomes were found to be 61 ± 1.8 and 83.56 ± 2.37%, respectively. These results may be due to the weak attachment of hydrophilic 5FU to the heads of the liposomal vesicles probably through adhesive force. Whereas chitosomes allow the rigid layer of chitosan to protect 5FU molecules attached to the surface, which increases EE%. The Entrapment efficiency increases with the coating of the liposomes, valuable explanation for this would be the binding capacity of the chitosan [43]. Moreover, electrostatic contact between negatively charged 5FU and positively charged chitosan results in an increase in the EE%. When compared to 5FU-chitosome, PEL improved the EE% (92.42 ± 1.69%), demonstrating CSSC’s effectiveness in reducing 5FU leakage from liposomes. CSSC surface modification of liposomes is facilitated by the development of a stiff layer of phospholipids. The outcomes were in good agreement with the study which developed PEG-liposomes coated with N-succinyl-chitosan and loaded with astaxanthin [29].

#### 3.5.2. Morphology, Particle Size, and Zeta Potential of 5FU-PEL

**Figure 8:**
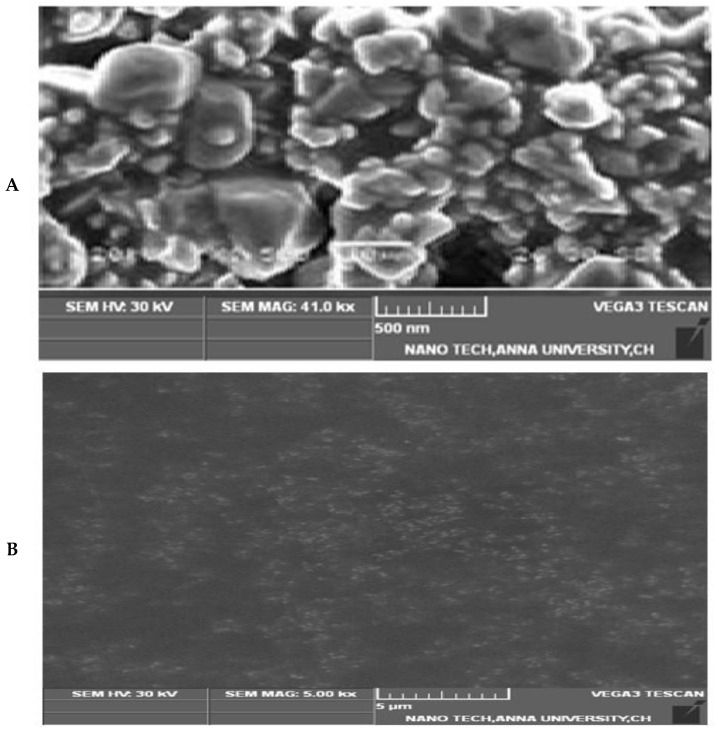
SEM Image of 5FU-PEL. (**A**) Show the surface morphology, (**B**) Show the loaded particles.

The morphology of 5FU-PEL was characterized by using SEM and the image is shown in Figure 8. CSSC-coated chitosome (PEL) samples appear to be made up of single or clustered impregnating vesicles. PEL revealed the presence of various clusters as well as individual vesicle forms is nearly spherical, possessing a smooth surface.

To validate the development of PEL, measurements of particle size and zeta potential have been taken. The particle size of 5FU-liposome was found to be 1.02 ± 0.06 μm and the zeta potential was measured at −19.3 mV while the 5FU-chitosome particle size was 9.3 ± 0.65 μm and the zeta potential transformed to the positive +29.7 mV. The results aligned with earlier findings that electrostatic interaction between the anionic charge of liposomes and the positive charge of chitosan resulted in the formation of chitosomes, having a positive electrical charge. CSSC-coated chitosomes (PEL) exhibited a negative zeta potential value of −18.56 mV and particle size increased to 10.5 ± 0.12 μm. Results revealed that CSSC adsorbs on the chitosomes’ surface, as one should predict that CSSC exists as a negatively charged polymer. Zeta potential is a crucial factor in understanding the electrostatic potential close to a nanoparticle’s surface and determining how stable it will be. Highly negative or positive zeta potentials increase the strength of the repelling forces, which prevent liposomes from naturally aggregating and allow for longer release of encapsulated 5FU in the colon [44,45].

#### 3.5.3. In-Vitro Drug Release Studies

**Figure 9:**
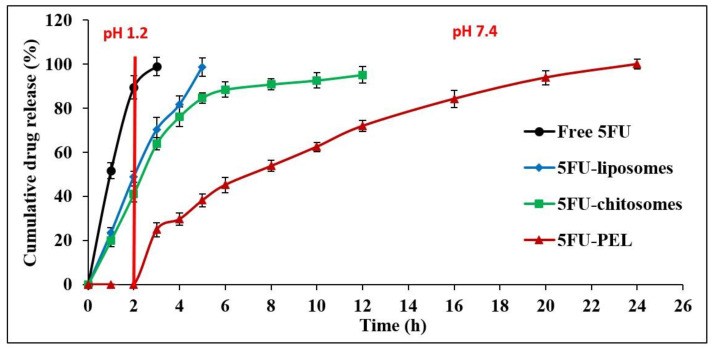
In vitro release profile of free 5FU, 5FU-liposomes, 5FU-chitosomes, and 5FU-PEL formulations.

The in vitro release profile of 5FU from liposomes, chitosomes, and PEL was investigated concerning the release of the free drug solution (control) by employing the dialysis bag technique under simulated gastrointestinal conditions. The 5FU aqueous solution demonstrated quick release, as anticipated and it was found 100% released at the end of 3 h. This result was aligned with the earlier study which demonstrated that 5FU is hydrophilic and triggered faster release at simulated gastric buffer [32]. The amount of 5FU released in this study through developed liposomal-based systems displayed a two-phase pattern, with a quick initial rate of drug release and a slow succeeding rate. A 5FU-liposomal formulation released 48.97% of the encapsulated 5FU rapidly in the early 2 h when placed in an acidic medium and after 4 h, 98% of 5FU was at basic condition. The 5FU can easily leak through the lipid membrane because it is a tiny, hydrophilic molecule [46]. The results indicated that the time needed for drug partitioning from the liposomes to the aqueous medium is thought to be a predominant stage in release rather than dissolution being the cause of the delayed release of 5FU from liposomes [47]. It was predicted that anionic membranes made of lipids would have some structural flaws due to increased electrostatic interactions that impact the permeability of the membrane, resulting in a faster diffusion process, hence, a total of 5FU being released. In-vitro release of chitosan-coated liposomes (chitosomes) revealed that 41.13% of 5FU was released in the first two hours at pH 1.2 due to great swellability in an acidic medium and extended the release in alkaline conditions. The result revealed that when liposomes were coated with chitosan, the release of 5-FU extended. Chitosan improved the stability and sustained release behavior of liposomes because of the proper 5FU encapsulation in the chitosan matrix in addition to its incorporation into the phospholipid bilayer [32,37]. Hence, drug release mechanisms from chitosomes are believed to involve both swelling and eroding of chitosan polymer and diffusion processes controlled by anionic lipids membrane [48]. As a result, chitosan dissolves through an acidic pH, and a significant portion of the drug is released in the stomach, which is not useful for targeting colon cancer. The release of drugs from CSSC-coated liposomes (PEL) has been investigated for the first 2 h at SGF as well as between 2 and 24 h with SIF while taking into account the average gastrointestinal transit time. Figure 9, shows that 5FU release increased significantly in SIF, which suggests the fact that currently produced PEL are capable of preventing drug release in SGF while releasing nearly the entire drug in the SIF medium. The explanation for this is that under the influence of acidic circumstances, the groups of carboxylic acids surrounding the liposomes reside in a nonionized state and happen to be hydrophilic below expectations, which could allow the drug to diffuse out from the system, and COOH groups in SIF act differently where strong ionization process may lead to extremely hydrophilic. It should be emphasized that comparable results have been obtained in swelling experiments [49,50]. CSSC-coated liposomes are also referred to as PEL due to their multiple electric charge density and swelling ability in basic conditions, which aids in drug release prolongation by producing an extensive gel layer by layer on the surface that covers the liposomes and ensuring stable uniform dispersion [51]. These properties, combined with a low level of toxicity, biodegradable in the body, and its capacity to help loosen the tightly coupled junctions of epithelial cells, facilitate the paracellular transport of drugs, thus rendering PEL an intriguing candidate for colon-specific drug delivery systems [45,52,53].

In-vitro release kinetics analysis was employed to calculate the 5FU release rate from PEL using various kinetic release models, including the zero-order, first-order, Higuchi, Korsemeyer–Peppas, and Hixson–Crowell models (Table 8). The drug 5FU attracted into the lipid membrane and covered by polyelectrolyte layers such as chitosan and CSSC may be the cause of the prolonged release phase, while the initial quicker release was attributed to the leaching of unentrapped 5FU adsorbed on the carriers’ outer surface. The result revealed that external CSSC polymers of these produced liposomal particles first expand, allowing fluids to permeate the chitosan layer. Another possible mechanism is that enclosed opposite charges polymers’ strong contacts with one another and the development of a tight layer atop anionic lipids could explain the prolonged release of 5FU from PEL. The Korsmeyer–Peppas model is a thorough explanation of the drug-releasing mechanism in use. The value of “n” dictates the manner of PEL release of the drug. If the value of n is greater than 0.45 but less than 0.89, the mechanism of drug release is Non-Fickian Diffusion (anomalous), if the value of n is greater than 0.89, the mechanism of drug release is followed after erosion of the polymer from the matrix (case II), and the anomalous mechanism of drug release includes both Non-Fickian Diffusion and erosion of the polymer used. A non-Fickian diffusion mechanism under colonic pH circumstances was supported by the optimized formulations and the n value was found larger than 0.45 (n = 0.6842). Thus, at colonic pH levels, supporting a non-Fickian diffusion process indicated swelling and erosion of the matrix system.

#### 3.5.4. Effect of pH on Swelling Characteristics of 5FU-PEL

**Figure 10:**
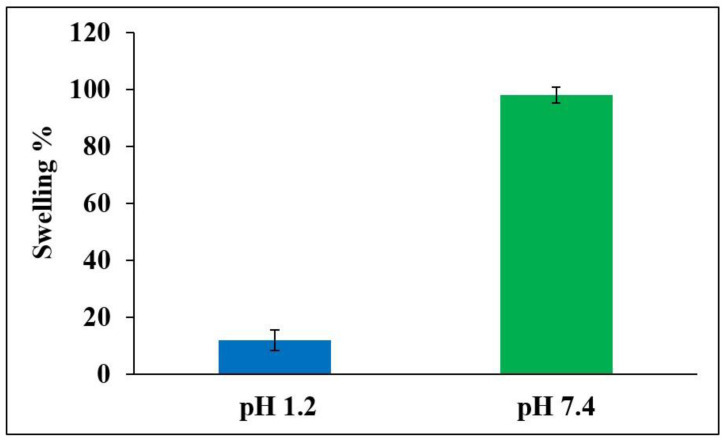
Swelling ability of optimized 5FU-PEL at pH 1.2 and pH 7.4 medium.

The influence of pH on the degree of swelling of 5FU-PEL is depicted in Figure 10. The swelling of PEL in 0.1N HCl and phosphate buffer saline was reported to be 12% and 98%, respectively. The findings clearly show that the swelling ability of PEL was decreased in acidic media. This could be due to carboxylic groups in the CSSC that exist in the nonionized form under acidic conditions and are poorly hydrophilic, whereas under basic conditions carboxylic groups exist in ionized form and are significantly hydrophilic [54]. As a result, 5FU encapsulated in PEL may be protected from the severe acidic environment of the stomach. The swelling of the PEL eventually increased to pH 7.4 after 6 h due to a rise in polymer swellability in basic pH, resulting in the relaxation of the polymeric network. In an alkaline medium, the PEL was expected to inflate upon liquid uptake and produce a hydrated, viscous layer surrounding the liposomes. The polymer inflated as a result of liquid entry into the vesicles, resulting in the creation of a rate-controlling membrane. CSSC, a hydrophilic polymer found in the chitosome’s outermost layer, is responsible for increased hydrophilicity. This suggests that CSSC may be efficiently attached to chitosomes.

#### 3.5.5. Mucoadhesive Properties of 5FU-PEL

**Figure 11:**
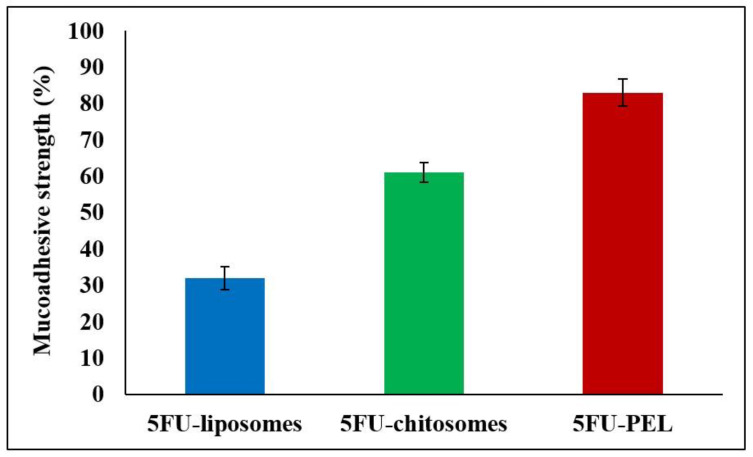
Mucoadhesive strength of 5FU-liposomes, 5FU-chitosomes, and 5FU-PEL formulations.

Figure 11, illustrates that the length of contact between PEL and the mucous membrane of the gastrointestinal tract impacts the work of adhesion. The interaction of mucoadhesive polymers with the mucus layer causes mucoadhesion, which is heavily dependent on the charge density and polymer structure. Depending on the polymer charge, positively charged polymers, such as chitosan, can combine with negatively charged mucins to form polyelectrolyte complexes and exhibit potent mucoadhesion [55]. The mucoadhesion of chitosan-coated liposomes (chitosomes) is higher in the stomach than in the duodenum, jejunum, and ileum [56]. This behavior might be caused by its high positive zeta potential, which permits strong mucoadhesion in the stomach in a short amount of time, and the rapid hydration of its chains resulting in the formation of thin gels that disintegrate easily [57]. PEL coated with CSSC could get beyond these restrictions that were seen in chitosomes. The negative zeta potential of PEL coated with CSSC resulted in weak mucoadhesion. The slower hydration of the coated polymer, on the other hand, encouraged the interpenetration of its chains with the mucus in the later part of the gastrointestinal tract, i.e. ileum and colon, at longer durations. According to Mura et al., slower hydration of the CSSC polymer and the encouragement of its chains to interact with mucus in the ileum and colon for prolonged periods could result in PEL having a high degree of mucoadhesivity [58]. The literature indicates that CSSC has a stronger mucoadhesion to colon mucosa than to stomach and small intestine mucosa. This is likely because the functional histology of the epithelia of small and large intestinal mucosa differs. At the tissue level, the absence of villi in the large intestine may be advantageous for mucoadhesion since it makes it easier for the PEL to adhere to the mucosa or epithelia [59].

### 3.6. Stability Study

Liposomes are inclined to be unstable due to several factors, including increased liposomal aggregation or fusion during storage. Additionally, drug leakage out of liposomes during storage can lower drug EE%. The size of the liposomes and the drug content were monitored at 25 °C/60% RH and 4 °C to ensure optimal liposomal function and therapeutic efficacy during the storage period. Table 9, shows that at room temperature and 4 °C, the 5FU-PEL formulation was stable for 1 and 6 months, respectively. The relative stability of the PEL formulation could be attributed to the electrostatic attraction force, and the process of CSSC coating, resulting in a thicker and more stable polymeric shell forming on the liposome surface, preventing vesicle fusion and aggregation during storage. This observation was supported by previous findings of quercetin and resveratrol-loaded liposomes coated with succinyl chitosan [44].

### 3.7. In Vitro Cytotoxicity Study

**Figure 12:**
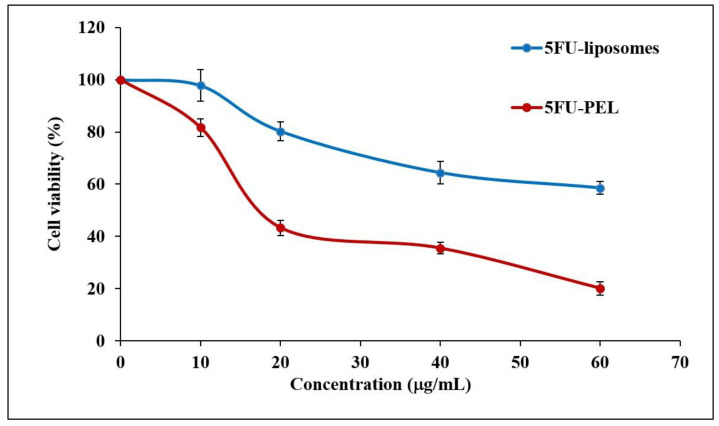
HT-29 colon cancer cell viability against 5FU-liposomes and 5FU-PEL.

In HT-29 cells, the antiproliferative effects of 5FU-liposomes and 5FU-PEL were examined over 24 h at concentrations of 10, 20, 40, and 60 μg/mL. Figure 12, shows the cell viability of colon cancer cells treated with 5FU-PEL and conventional 5FU-liposomes. Results from the MTT experiment indicated that PEL treatment of the HT-29 cells resulted in significantly lower cell viability (*p* < 0.05) as compared to treatment with conventional liposomes. The findings suggested that the PEL increases the permeability of 5FU, offering maximum drug exposure to the cancerous cells and, as a result, significantly inhibits colon cancer cell proliferation. Interestingly, the pharmacological action of 5FU is diminished relatively quickly after absorption due to its short half-life [60]. Our results suggest that 5FU-PEL allows it to adhere to the cell’s surface first before achieving sustained drug release at the colon pH region and then enhanced membrane permeability, providing a potential PEL to improve its anticancer efficacy.

### 3.8. In Vivo Pharmacokinetic Parameters

**Figure 13:**
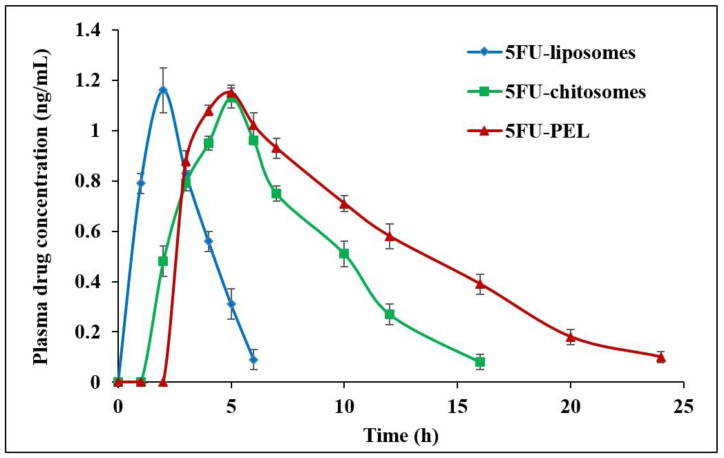
In vivo plasma drug concentration after administration of 5FU-liposomes, 5FU-chitosomes, and 5FU-PEL formulations.

The effect of plasma drug concentration after administration of 5FU-liposomes, 5FU-chitosomes, and 5FU-PEL formulations is shown in Figure 13. The pharmacokinetics parameters of single-dose administration of 5FU with three different formulations were calculated and presented in Table 10. The t_max_ of the 5FU-loaded chitosomes and PEL was significantly different (*p* < 0.05) from that of the 5FU-liposomes. A low t_max_ value for the 5FU-liposomes (2 h) indicates rapid absorption while the higher t_max_ of the 5FU-PEL (5 h) suggests slower absorption. This delayed absorption of PEL is most likely due to the sustained release of the drug. On the other hand, the C_max_ of 5FU-PEL was not significantly different from the 5FU-liposomes. The half-life of the 5FU-liposomes was low which indicates rapid removal of the drug from plasma. On the other hand, the 5FU-PEL formulation exhibited higher half-life and low elimination rate constant values indicating slower drug disposition and prolonged effect. These results evidenced the ability of CSSC to enhance the absorption of 5FU at the colon region. The mean residence time (MRT) of PEL (10.445 ± 1.57 h) was significantly higher than chitosomes (7.243 ± 1.18 h) with a *p*-value < 0.05. This result revealed that PEL may enhance mucoadhesive properties and membrane permeation ability which could help to maintain plasma drug concentration for a longer time [19].

It was found that AUC_0−∞_ for 5FU-liposomes, 5FU-chitosomes, and 5FU-PEL were 3.819 ± 0.65, 8.543 ± 1.02, and 12.763 ± 1.78 ng·h/mL, respectively. The relative bioavailability % of 5FU-chitosomes and 5FU-PEL were 223.69 ± 3.82% and 334.19 ± 2.07%, respectively. These results may be due to the CSSC coat which contains -COOH groups, having the potential to chelate Ca2+ ions present in adherens junctions (AJs) and cause disruption of epithelial tight junctions (TJs) in the jejunum and ileum. When compared to 5FU-chitosomes, the synergistic actions of PEL facilitate a significant improvement in the oral bioavailability of 5FU [61].

## 4. Conclusions

The authors concluded that 5FU can be successfully prepared as PEL. The central composite design was able to obtain an optimized formulation of 5FU-chitosomes. The optimized formulation was prepared with 40.9485 mg of cholesterol, 13.1171 mg of chitosan, and 5.30644 mg of surfactant and exhibited high EE% and prolonged drug release. PEL limited the release of 5FU in the gastric medium however prolonged the drug release in the colon site. A MTT assay study revealed that PEL treatment significantly reduced the viability of HT-29 cells. The 5FU-PEL improved the in vivo pharmacokinetics when compared to 5FU-liposome and 5FU-chitosome. PEL increases 5FU permeability, which limits colon cancer cell proliferation and allows high drug concentrations to penetrate cells.

## Figures and Tables

**Table 1:** Formulation factors for preparation of 5FU-chitosomes and corresponding levels.

Independent Variable	Variable Levels				
	−α	−1	0	+1	+α
X_1_: Cholesterol amount (mg)	19.3	32.6	45.9	59.2	72.5
X_2_: Chitosan amount (mg)	3	8	13	18	23
X_3_: Surfactant amount (mg)	1	3	5	7	9
**Responses**	**Constraints**				
Y_1_: Entrapment efficiency (%)	Maximize				
Y_2_: Particle size (µm)	Minimize				
Y_3_: Drug release Q_12 h_ (%)	>90%				

**Table 2:** Outline of Central composite design with the results of responses on three independent factors.

	Factor 1	Factor 2	Factor 3	Response 1	Response 2	Response 3
Run	A: Cholesterol	B: Chitosan	C: Surfactant	EE (%)	Size (µm)	Drug Release (Q_12 h_) %
1	0	0	0	89 ± 2.02	11.6 ± 0.4	94.75 ± 4.5
2	1	−1	1	36 ± 1.2	14.6 ± 0.8	99.89 ± 2.4
3	1	1	−1	53 ± 2.56	29.4 ± 0.1	67.31 ± 3.7
4	0	0	0	86.32 ± 1.56	12.9 ± 0.2	96.52 ± 2.8
5	1.68179	0	0	53 ± 2.89	27.8 ± 0.3	92.36 ± 5.1
6	−1.68179	0	0	60 ± 1.00	10.5 ± 0.4	98.24 ± 3.9
7	0	0	−1.68179	78 ± 2.44	32.4 ± 0.5	60.25 ± 2.6
8	−1	1	−1	78 ± 2.50	19.8 ± 0.7	67.12 ± 3.8
9	0	0	0	88.15 ± 3.2	10.65 ± 0.6	93.17 ± 3.6
10	1	−1	−1	64 ± 1.37	31.7 ± 0.7	85.69 ± 2.7
11	0	0	0	83.27 ± 2.2	15.03 ± 0.5	91.08 ± 3.4
12	0	0	1.68179	41 ± 3.40	4.02 ± 0.6	75.21 ± 4.2
13	1	1	1	76 ± 1.98	12.68 ± 0.2	89.41 ± 5.3
14	0	−1.68179	0	30 ± 3.72	13.6 ± 0.3	99.34 ± 4.9
15	−1	1	1	48 ± 3.01	9.1 ± 0.9	61.32 ± 3.8
16	0	0	0	85.17 ± 2.45	12.37 ± 0.4	92.78 ± 4.1
17	0	1.68179	0	55.4 ± 1.05	22.7 ± 0.2	69.32 ± 2.8
18	−1	−1	−1	78 ± 1.67	14.1 ± 0.7	80.21 ± 3.6
19	−1	−1	1	20 ± 1.88	3.4 ± 0.2	85.64 ± 4.5
20	0	0	0	80.14 ± 1.23	11.9 ± 0.6	80.95 ± 3.7

**Table 3:** Model summary statistics for EE, Size, and DR (response Y1, Y2, and Y3) of 5FU-chitosomes.

Source	SD	R^2^	Adjusted R^2^	Predicted R^2^	Press	Comment
**Y_1_ (EE)**						
Quadratic	4.99	0.9707	0.9443	0.8546	1663.99	Suggested
Cubic	5.12	0.9815	0.9415	−1.6899	22,893.44	Aliased
**Y_2_ (Size)**						
Quadratic	2.07	0.9690	0.9411	0.8144	256.92	Suggested
Cubic	2.01	0.9825	0.9446	−1.0977	2904.49	Aliased
**Y_3_ (DR_12 h_)**						
Quadratic	6.44	0.9718	0.9564	0.8925	2290.50	Suggested
Cubic	5.31	0.9478	0.9347	−1.2463	4034.59	Aliased

**Table 4:** ANOVA for quadratic model for EE (response Y_1_) of 5FU-chitosomes.

Source	Sum of Squares	df	Mean Square	F-Value	*p*-Value	
**Model**	8261.66	9	917.96	36.81	<0.0001	Significant
A-Cholesterol	3.36	1	3.36	0.1347	0.7213	
B-Chitosan	728.10	1	728.10	29.19	0.0003	
C-Surfactant	1764.33	1	1764.33	70.74	<0.0001	
AB	0.1250	1	0.1250	0.0050	0.9450	
AC	861.13	1	861.13	34.53	0.0002	
BC	780.13	1	780.13	31.28	0.0002	
A^2^	1166.21	1	1166.21	46.76	<0.0001	
B^2^	2774.30	1	2774.30	111.24	<0.0001	
C^2^	907.41	1	907.41	36.38	0.0001	
**Residual**	249.40	10	24.94			
Lack of Fit	195.80	5	39.16	3.65	0.0907	not significant
Pure Error	53.61	5	10.72			
**Cor Total**	8511.07	19				

**Table 5:** ANOVA for quadratic model for Size (response Y_2_) of 5FU-chitosomes.

Source	Sum of Squares	df	Mean Square	F-Value	*p*-Value	
**Model**	1341.67	9	149.07	34.74	<0.0001	Significant
A-Cholesterol	369.90	1	369.90	86.19	<0.0001	
B-Chitosan	37.02	1	37.02	8.63	0.0149	
C-Surfactant	776.06	1	776.06	180.83	<0.0001	
AB	30.50	1	30.50	7.11	0.0237	
AC	19.28	1	19.28	4.49	0.0601	
BC	0.0180	1	0.0180	0.0042	0.9496	
A^2^	55.14	1	55.14	12.85	0.0050	
B^2^	37.01	1	37.01	8.62	0.0149	
C^2^	38.00	1	38.00	8.85	0.0139	
**Residual**	42.92	10	4.29			
Lack of Fit	31.80	5	6.36	2.86	0.1368	not significant
Pure Error	11.12	5	2.22			
**Cor Total**	1384.59	19				

**Table 6:** ANOVA for quadratic model for DR_12 h_ (response Y_3_) of of 5FU-chitosomes.

Source	Sum of Squares	Df	Mean Square	F-Value	*p*-Value	
**Model**	2822.18	9	313.58	7.55	0.0020	Significant
A-Cholesterol	106.41	1	106.41	2.56	0.1404	
B-Chitosan	998.20	1	998.20	24.05	0.0006	
C-Surfactant	273.27	1	273.27	6.58	0.0281	
AB	9.14	1	9.14	0.2201	0.6490	
AC	168.09	1	168.09	4.05	0.0719	
BC	1.39	1	1.39	0.0334	0.8587	
A^2^	10.11	1	10.11	0.2435	0.6324	
B^2^	133.27	1	133.27	3.21	0.1034	
C^2^	1144.09	1	1144.09	27.56	0.0004	
**Residual**	415.08	10	41.51			
Lack of Fit	263.42	5	52.68	1.74	0.2796	not significant
Pure Error	151.66	5	30.33			
**Cor Total**	3237.26	19				

**Table 7:** Experimental and predicted values of responses for the optimized 5FU-chitosomes.

Point Prediction	Entrapment Efficiency (%)	Particle Size (μm)	Drug Release Q_12 h_ (%)
**Predicted**	85.221	9.07	93.72
**Observed**	83.56 ± 2.37	9.3 ± 0.65	95.16 ± 3.74
**% error**	−1.94	2.53	1.54

% error = (observed value-predicted value)/predicted value × 100.

**Table 8:** In-vitro release kinetics model of 5FU-PEL formulation.

Parameter	Zero Order	First Order	Higuchi Model	Krosmeyer Peppa’s Model	Hixson Crowel’s Cube Root Model	‘n’
K	4.4478	0.0582	24.661	1.0939	0.0797	0.6842
R^2^	0.914	0.9724	0.9927	0.9859	0.898	

**Table 9:** Stability report of 5FU-PEL at room temperature and 4 °C.

Storage Temperature Condition	Particle Size (μm)		Drug Content (%)	
	Initial	After Six Months	Initial	After Six Months
4 °C	10.5 ± 0.12	10.9 ± 0.98	98.56 ± 2.37	96.23 ± 1.86
25 °C/60% RH		11.67 ± 0.82		94.61 ± 2.40

**Table 10:** Effect of different formulations on pharmacokinetics parameters.

Pharmacokinetics Parameters	5FU-Liposomes	5FU-Chitosomes	5FU-PEL
C_max_ (ng/mL)	1.16 ± 0.16	1.13 ± 0.19	1.15 ± 0.07
T_max_(h)	2	5	5
AUC_0-∞_ (ng·h/mL)	3.819 ± 0.65	8.543 ± 1.02	12.763 ± 1.78
K_el_ (h^−1^)	0.725 ± 0.06	0.243 ± 0.03	0.128 ± 0.01
t_1/2_ (h)	0.955 ± 0.03	2.846 ± 0.39	5.3938 ± 0.94
MRT (h)	2.724 ± 0.23	7.243 ± 1.18	10.445 ± 1.57
Relative Bioavailability (%)	_	223.69 ± 3.82	334.19 ± 2.07 *

Results are expressed as mean ± s.d., n = 6. * *p* < 0.05, 5FU-PEL versus 5FU-Chitosomes.

## Data Availability

Data are available from the corresponding author upon request.

## References

[1] Wang S., Chen Y., Guo J., Huang Q. (2023). Liposomes for Tumor Targeted Therapy: A Review. Int. J. Mol. Sci..

[2] Sharifi-Azad M., Fathi M., Cho W.C., Barzegari A., Dadashi H., Dadashpour M., Jahanban-Esfahlan R. (2022). Recent Advances in Targeted Drug Delivery Systems for Resistant Colorectal Cancer. Cancer Cell Int..

[3] Sercombe L., Veerati T., Moheimani F., Wu S.Y., Sood A.K., Hua S. (2015). Advances and Challenges of Liposome Assisted Drug Delivery. Front. Pharmacol..

[4] Assadpour S., Akhtari J., Shiran M.R. (2022). Pharmacokinetics Study of Chitosan-Coated Liposomes Containing Sumatriptan in the Treatment of Migraine. Casp. J. Intern. Med..

[5] Kim H.Y., Cheon J.H., Lee S.H., Min J.Y., Back S.-Y., Song J.G., Kim D.H., Lim S.-J., Han H.-K. (2020). Ternary Nanocomposite Carriers Based on Organic Clay-Lipid Vesicles as an Effective Colon-Targeted Drug Delivery System: Preparation and in Vitro/in Vivo Characterization. J. Nanobiotechnol..

[6] Wang C., Han Z., Wu Y., Lu X., Tang X., Xiao J., Li N. (2021). Enhancing Stability and Anti-Inflammatory Properties of Curcumin in Ulcerative Colitis Therapy Using Liposomes Mediated Colon-Specific Drug Delivery System. Food Chem. Toxicol..

[7] Liu Y., Li X., Pen R., Zuo W., Chen Y., Sun X., Gou J., Guo Q., Wen M., Li W. (2022). Targeted Delivery of Irinotecan to Colon Cancer Cells Using Epidermal Growth Factor Receptor-Conjugated Liposomes. BioMed. Eng. OnLine.

[8] Sebaaly C., Trifan A., Sieniawska E., Greige-Gerges H. (2021). Chitosan-Coating Effect on the Characteristics of Liposomes: A Focus on Bioactive Compounds and Essential Oils: A Review. Processes.

[9] Takeuchi H., Matsui Y., Sugihara H., Yamamoto H., Kawashima Y. (2005). Effectiveness of Submicron-Sized, Chitosan-Coated Liposomes in Oral Administration of Peptide Drugs. Int. J. Pharm..

[10] Andersen T., Vanić Ž., Flaten G., Mattsson S., Tho I., Škalko-Basnet N. (2013). Pectosomes and Chitosomes as Delivery Systems for Metronidazole: The One-Pot Preparation Method. Pharmaceutics.

[11] Xian J., Zhong X., Gu H., Wang X., Li J., Li J., Wu Y., Zhang C., Zhang J. (2021). Colonic Delivery of Celastrol-Loaded Layer-by-Layer Liposomes with Pectin/Trimethylated Chitosan Coating to Enhance Its Anti-Ulcerative Colitis Effects. Pharmaceutics.

[12] Wang G., Yang Y., Yi D., Yuan L., Yin P.-H., Ke X., Jun-Jie W., Tao M.-F. (2022). Eudragit S100 Prepared pH-Responsive Liposomes-Loaded Betulinic Acid against Colorectal Cancer In Vitro and In Vivo. J. Liposome Res..

[13] Alghurabi H., Tagami T., Ogawa K., Ozeki T. (2022). Preparation, Characterization and In Vitro Evaluation of Eudragit S100-Coated Bile Salt-Containing Liposomes for Oral Colonic Delivery of Budesonide. Polymers.

[14] Jubeh T.T., Barenholz Y., Rubinstein A. (2004). Differential Adhesion of Normal and Inflamed Rat Colonic Mucosa by Charged Liposomes. Pharm. Res..

[15] Werle M., Takeuchi H. (2009). Chitosan–Aprotinin Coated Liposomes for Oral Peptide Delivery: Development, Characterisation and in Vivo Evaluation. Int. J. Pharm..

[16] Barea M.J., Jenkins M.J., Gaber M.H., Bridson R.H. (2010). Evaluation of Liposomes Coated with a pH Responsive Polymer. Int. J. Pharm..

[17] Barea M.J., Jenkins M.J., Lee Y.S., Johnson P., Bridson R.H. (2012). Encapsulation of Liposomes within pH Responsive Microspheres for Oral Colonic Drug Delivery. Int. J. Biomater..

[18] Li R., Deng L., Cai Z., Zhang S., Wang K., Li L., Ding S., Zhou C. (2017). Liposomes Coated with Thiolated Chitosan as Drug Carriers of Curcumin. Mater. Sci. Eng. C.

[19] Ubaidulla U., Khar R.K., Ahmad F.J., Sultana Y., Panda A.K. (2007). Development and Characterization of Chitosan Succinate Microspheres for the Improved Oral Bioavailability of Insulin. J. Pharm. Sci..

[20] Ubaidulla U., Khar R.K., Ahmed F.J., Panda A.K. (2007). Development and In-Vivo Evaluation of Insulin-Loaded Chitosan Phthalate Microspheres for Oral Delivery. J. Pharm. Pharmacol..

[21] Karuna D., Ubaidulla U., Rathnam G., Mani G., Jang H.T., Mani G. (2016). Preparation And Evaluatıon Of Chitosan Succinate Pellets Using Extrusion-Spheronization Technology: Processing And In Vitro Characterization. Turk. J. Pharm. Sci..

[22] Sinha P., Udhumansha U., Rathnam G., Ganesh M., Jang H.T. (2018). Capecitabine Encapsulated Chitosan Succinate-Sodium Alginate Macromolecular Complex Beads for Colon Cancer Targeted Delivery: In Vitro Evaluation. Int. J. Biol. Macromol..

[23] Janardhanam L.S.L., Deokar A.S., Bollareddy S.R., Venuganti V.V.K. (2022). Colon-Targeted Layer-by-Layer Self-Assembled Film: Pharmacokinetic Analysis of BCS Class I and Class III Model Drugs. AAPS PharmSciTech.

[24] Ibrahim B., Mady O.Y., Tambuwala M.M., Haggag Y.A. (2022). pH-Sensitive Nanoparticles Containing 5-Fluorouracil and Leucovorin as an Improved Anti-Cancer Option for Colon Cancer. Nanomedicine.

[25] Wang J., Gong J., Wei Z. (2022). Strategies for Liposome Drug Delivery Systems to Improve Tumor Treatment Efficacy. AAPS PharmSciTech.

[26] Krajewska J.B., Bartoszek A., Fichna J. (2018). New Trends in Liposome-Based Drug Delivery in Colorectal Cancer. Mini-Rev. Med. Chem..

[27] El-Alfy E.A., El-Bisi M.K., Taha G.M., Ibrahim H.M. (2020). Preparation of Biocompatible Chitosan Nanoparticles Loaded by Tetracycline, Gentamycin and Ciprofloxacin as Novel Drug Delivery System for Improvement the Antibacterial Properties of Cellulose Based Fabrics. Int. J. Biol. Macromol..

[28] Ritger P.L., Peppas N.A. (1987). A Simple Equation for Description of Solute Release II. Fickian and Anomalous Release from Swellable Devices. J. Control. Release.

[29] Liu A., Chai X., Zhu S., Chin P., He M., Xu Y.-J., Liu Y. (2023). Effects of N-Succinyl-Chitosan Coating on Properties of Astaxanthin-Loaded PEG-Liposomes: Environmental Stability, Antioxidant/Antibacterial Activities, and in Vitro Release. Int. J. Biol. Macromol..

[30] Qushawy M. (2021). Effect of the Surfactant and Liquid Lipid Type in the Physico-Chemical Characteristics of Beeswax-Based Nanostructured Lipid Carrier (NLC) of Metformin. Pharm. Nanotechnol..

[31] Correa S., Boehnke N., Deiss-Yehiely E., Hammond P.T. (2019). Solution Conditions Tune and Optimize Loading of Therapeutic Polyelectrolytes into Layer-by-Layer Functionalized Liposomes. ACS Nano.

[32] Alomrani A., Badran M., Harisa G.I., ALshehry M., Alhariri M., Alshamsan A., Alkholief M. (2019). The Use of Chitosan-Coated Flexible Liposomes as a Remarkable Carrier to Enhance the Antitumor Efficacy of 5-Fluorouracil against Colorectal Cancer. Saudi Pharm. J..

[33] Uthumansha U., Prabahar K., Gajapathy D.B., El-Sherbiny M., Elsherbiny N., Qushawy M. (2023). Optimization and In Vitro Characterization of Telmisartan Loaded Sodium Alginate Beads and Its In Vivo Efficacy Investigation in Hypertensive Induced Animal Model. Pharmaceutics.

[34] Alghazwani Y., Venkatesan K., Prabahar K., El-Sherbiny M., Elsherbiny N., Qushawy M. (2023). The Combined Anti-Tumor Efficacy of Bioactive Hydroxyapatite Nanoparticles Loaded with Altretamine. Pharmaceutics.

[35] Mukhopadhyay P., Maity S., Mandal S., Chakraborti A.S., Prajapati A.K., Kundu P.P. (2018). Preparation, Characterization and in Vivo Evaluation of pH Sensitive, Safe Quercetin-Succinylated Chitosan-Alginate Core-Shell-Corona Nanoparticle for Diabetes Treatment. Carbohydr. Polym..

[36] Nguyen T.X., Huang L., Liu L., Elamin Abdalla A.M., Gauthier M., Yang G. (2014). Chitosan-Coated Nano-Liposomes for the Oral Delivery of Berberine Hydrochloride. J. Mater. Chem. B.

[37] Badran M.M., Alomrani A.H., Almomen A., Bin Jardan Y.A., Abou El Ela A.E.S. (2022). Novel Metoprolol-Loaded Chitosan-Coated Deformable Liposomes in Thermosensitive In Situ Gels for the Management of Glaucoma: A Repurposing Approach. Gels.

[38] Channarong S., Chaicumpa W., Sinchaipanid N., Mitrevej A. (2011). Development and Evaluation of Chitosan-Coated Liposomes for Oral DNA Vaccine: The Improvement of Peyer’s Patch Targeting Using a Polyplex-Loaded Liposomes. AAPS PharmSciTech.

[39] Sriwidodo, Umar A.K., Wathoni N., Zothantluanga J.H., Das S., Luckanagul J.A. (2022). Liposome-Polymer Complex for Drug Delivery System and Vaccine Stabilization. Heliyon.

[40] Ingvarsson P.T., Yang M., Mulvad H., Nielsen H.M., Rantanen J., Foged C. (2013). Engineering of an Inhalable DDA/TDB Liposomal Adjuvant: A Quality-by-Design Approach towards Optimization of the Spray Drying Process. Pharm. Res..

[41] Bayuo J., Abukari M.A., Pelig-Ba K.B. (2020). Optimization Using Central Composite Design (CCD) of Response Surface Methodology (RSM) for Biosorption of Hexavalent Chromium from Aqueous Media. Appl. Water Sci..

[42] Zeng C., Jiang W., Tan M., Yang X., He C., Huang W., Xing J. (2016). Optimization of the Process Variables of Tilianin-Loaded Composite Phospholipid Liposomes Based on Response Surface-Central Composite Design and Pharmacokinetic Study. Eur. J. Pharm. Sci..

[43] Yang Z., Liu J., Gao J., Chen S., Huang G. (2015). Chitosan Coated Vancomycin Hydrochloride Liposomes: Characterizations and Evaluation. Int. J. Pharm..

[44] Caddeo C., Pons R., Carbone C., Fernàndez-Busquets X., Cardia M.C., Maccioni A.M., Fadda A.M., Manconi M. (2017). Physico-Chemical Characterization of Succinyl Chitosan-Stabilized Liposomes for the Oral Co-Delivery of Quercetin and Resveratrol. Carbohydr. Polym..

[45] Graisuwan W., Wiarachai O., Ananthanawat C., Puthong S., Soogarun S., Kiatkamjornwong S., Hoven V.P. (2012). Multilayer Film Assembled from Charged Derivatives of Chitosan: Physical Characteristics and Biological Responses. J. Colloid Interface Sci..

[46] Hou J., Li C., Cheng L., Guo S., Zhang Y., Tang T. (2011). Study on Hydrophilic 5-Fluorouracil Release from Hydrophobic Poly(ϵ-Caprolactone) Cylindrical Implants. Drug Dev. Ind. Pharm..

[47] Abbas H., El-Feky Y.A., Al-Sawahli M.M., EL-Deeb N.M., El-Nassan H.B., Zewail M. (2022). Development and Optimization of Curcumin Analog Nano-Bilosomes Using 2^1^.3^1^ Full Factorial Design for Anti-Tumor Profiles Improvement in Human Hepatocellular Carcinoma: In-Vitro Evaluation, In-Vivo Safety Assay. Drug Deliv..

[48] Beenken K.E., Smith J.K., Skinner R.A., Mclaren S.G., Bellamy W., Gruenwald M.J., Spencer H.J., Jennings J.A., Haggard W.O., Smeltzer M.S. (2014). Chitosan Coating to Enhance the Therapeutic Efficacy of Calcium Sulfate-Based Antibiotic Therapy in the Treatment of Chronic Osteomyelitis. J. Biomater. Appl..

[49] Yan C., Chen D., Gu J., Hu H., Zhao X., Qiao M. (2006). Preparation of N-Succinyl-Chitosan and Its Physical-Chemical Properties as a Novel Excipient. Yakugaku Zasshi.

[50] Bashir S., Teo Y.Y., Ramesh S., Ramesh K., Khan A.A. (2015). N-Succinyl Chitosan Preparation, Characterization, Properties and Biomedical Applications: A State of the Art Review. Rev. Chem. Eng..

[51] Mura C., Manconi M., Valenti D., Manca M.L., Díez-Sales O., Loy G., Fadda A.M. (2011). In Vitro Study of N-Succinyl Chitosan for Targeted Delivery of 5-Aminosalicylic Acid to Colon. Carbohydr. Polym..

[52] Wang Y., Tu S., Li R., Yang X., Liu L., Zhang Q. (2010). Cholesterol Succinyl Chitosan Anchored Liposomes: Preparation, Characterization, Physical Stability, and Drug Release Behavior. Nanomed. Nanotechnol. Biol. Med..

[53] Seong J.S., Yun M.E., Park S.N. (2018). Surfactant-Stable and pH-Sensitive Liposomes Coated with N-Succinyl-Chitosan and Chitooligosaccharide for Delivery of Quercetin. Carbohydr. Polym..

[54] Ahmed T., Aljaeid B. (2016). Preparation, Characterization, and Potential Application of Chitosan, Chitosan Derivatives, and Chitosan Metal Nanoparticles in Pharmaceutical Drug Delivery. Drug Des. Dev. Ther..

[55] Shin G.H., Chung S.K., Kim J.T., Joung H.J., Park H.J. (2013). Preparation of Chitosan-Coated Nanoliposomes for Improving the Mucoadhesive Property of Curcumin Using the Ethanol Injection Method. J. Agric. Food Chem..

[56] Athavale R., Sapre N., Rale V., Tongaonkar S., Manna G., Kulkarni A., Shirolkar M.M. (2022). Tuning the Surface Charge Properties of Chitosan Nanoparticles. Mater. Lett..

[57] Manconi M., Nácher A., Merino V., Merino-Sanjuan M., Manca M.L., Mura C., Mura S., Fadda A.M., Diez-Sales O. (2013). Improving Oral Bioavailability and Pharmacokinetics of Liposomal Metformin by Glycerolphosphate–Chitosan Microcomplexation. AAPS PharmSciTech.

[58] Mura C., Nácher A., Merino V., Merino-Sanjuán M., Manconi M., Loy G., Fadda A.M., Díez-Sales O. (2012). Design, Characterization and in Vitro Evaluation of 5-Aminosalicylic Acid Loaded N-Succinyl-Chitosan Microparticles for Colon Specific Delivery. Colloids Surf. B Biointerfaces.

[59] Thirawong N., Nunthanid J., Puttipipatkhachorn S., Sriamornsak P. (2007). Mucoadhesive Properties of Various Pectins on Gastrointestinal Mucosa: An in Vitro Evaluation Using Texture Analyzer. Eur. J. Pharm. Biopharm..

[60] Goirand F., Lemaitre F., Launay M., Tron C., Chatelut E., Boyer J.-C., Bardou M., Schmitt A. (2018). How Can We Best Monitor 5-FU Administration to Maximize Benefit to Risk Ratio?. Expert. Opin. Drug Metab. Toxicol..

[61] Tan C., Wang J., Sun B. (2021). Biopolymer-Liposome Hybrid Systems for Controlled Delivery of Bioactive Compounds: Recent Advances. Biotechnol. Adv..

